# Skull shape and size variation within and between *mendocinus* and *torquatus* groups in the genus *Ctenomys* (Rodentia: Ctenomyidae) in chromosomal polymorphism context

**DOI:** 10.1590/1678-4685-GMB-2017-0074

**Published:** 2018

**Authors:** Rodrigo Fornel, Pedro Cordeiro-Estrela, Thales Renato O. de Freitas

**Affiliations:** 1 Departamento de Genética, Universidade Federal do Rio Grande do Sul, Porto Alegre, RS, Brazil; 2 Universidade Regional Integrada do Alto Uruguai e das Missões – Campus de Erechim, Erechim, RS, Brazil; 3 Departamento de Sistemática e Ecologia, Centro de Ciências Exatas e da Natureza – Campus I, Universidade Federal da Paraíba, João Pessoa, PB, Brazil; 4 Programa de Pós-Graduação em Genética e Biologia Molecular, Universidade Federal do Rio Grande do Sul, Porto Alegre, RS, Brazil

**Keywords:** Cranium, geometric morphometrics, phenotypic evolution, subterranean rodent

## Abstract

We tested the association between chromosomal polymorphism and skull shape and size variation in two groups of the subterranean rodent *Ctenomys*. The hypothesis is based on the premise that chromosomal rearrangements in small populations, as it occurs in *Ctenomys*, produce reproductive isolation and allow the independent diversification of populations. The *mendocinus* group has species with low chromosomal diploid number variation (2n=46-48), while species from the *torquatus* group have a higher karyotype variation (2n=42-70). We analyzed the shape and size variation of skull and mandible by a geometric morphometric approach, with univariate and multivariate statistical analysis in 12 species from *mendocinus* and *torquatus* groups of the genus *Ctenomys*. We used 763 adult skulls in dorsal, ventral, and lateral views, and 515 mandibles in lateral view and 93 landmarks in four views. Although we expected more phenotypic variation in the *torquatus* than the *mendocinus* group, our results rejected the hypothesis of an association between chromosomal polymorphism and skull shape and size variation. Moreover, the *torquatus* group did not show more variation than *mendocinus.* Habitat heterogeneity associated to biomechanical constraints and other factors like geography, phylogeny, and demography, may affect skull morphological evolution in *Ctenomys*.

## Introduction

The genus *Ctenomys* is composed of approximately 70 species that are found in South America (Bidau, 2015; Freitas, 2016). These subterranean rodents show the largest chromosomal polymorphism among mammals, with diploid numbers varying from 2n=10 in *C. steinbachi* to 2n=70 in *C. pearsoni* (Reig *et al.*, 1990; Ortells and Barrantes, 1994). Because of this large karyotype variation, chromosomal speciation has been proposed as a probable, or primary mechanism of cladogenesis within the genus *Ctenomys* (King, 1993; Ortells and Barrantes, 1994). Adaptive radiation caused by key innovations to the underground niche (Nevo, 1979) and patchy population structure (Reig *et al.*, 1990) have been proposed as alternative or concurrent mechanisms to explain high rates of diversification. However, most of these mechanisms have been seriously challenged by analyses based on molecular data. The mere fact that *Ctenomys* presents high rates of diversification has failed to receive significant support when compared to *Hystricognathous* sister lineages (Cook and Lessa, 1998). Tomasco and Lessa (2007) have shown that chromosomal populations are polyphyletic relative to mitochondrial DNA in *C. pearsoni*. Adding to this fact, no sign of negative heterosis has been found in hybrid zones of *Ctenomys* (Freitas, 1997; Gava and Freitas, 2002, 2003). Negative heterosis would be required in traditional models of chromosomal speciation to disrupt gene flow between populations. Thus, its absence seriously undermines these traditional models as a primary mechanism of diversification in *Ctenomys*. However, Navarro and Barton (2003) and Rieseberg and Livingstone (2003) have proposed that the reduced recombination of rearranged chromosomes might favor the accumulation of adaptive differences on rearranged regions. In this article, we analyze an adaptive structure, the skull, within two clades of *Ctenomys* that differ radically in number of chromosomal rearrangements.

Studies based on morphological, cytogenetic, and molecular data have proposed different lineages or main groups within the genus *Ctenomys* (Lessa and Cook, 1998; Contreras and Bidau, 1999; D’Elia *et al.*, 1999; Mascheretti *et al.*, 2000; Slamovits *et al.*, 2001; Parada *et al.*, 2011). Two of these groups, *mendocinus* and *torquatus*, are very different in chromosomal polymorphism.

**Figure 1:**
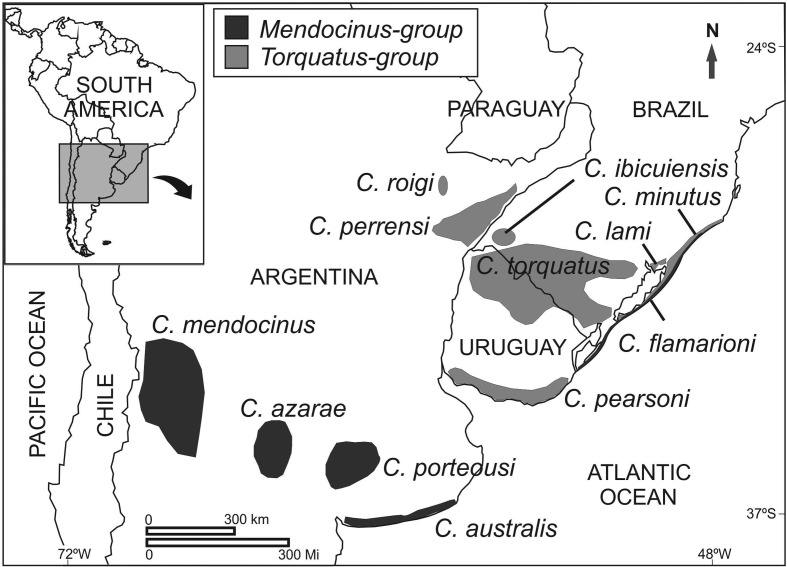
Map with distribution of 12 *Ctenomys* species belonging to the *mendocinus* and *torquatus* groups, with the exception of *C. rionegrensis* and *C. chasiquensis*. Mendocinus-group in black: *C. flamarioni* (2n=48), *C. australis* (2n=48), *C. porteousi* (2n=47-48), *C. azarae* (2n=46-48), and *C. mendocinus* (2n=47-48). Torquatus-group in grey: *C. minutus* (2n=42-50), *C. lami* (2n=54-58), *C. torquatus* (2n=40-46), *C. pearsoni* (2n=56-70), *C. perrensi* (2n=50-58), *C. ibicueisis* (2n=50) and *C. roigi* (2n=48).

The *mendocinus* group, suggested by Massarini *et al.* (1991), is known for its low variation in chromosomal diploid number. The majority of species have from 2n=46 to 2n=48, the exception being *C. rionegrensis* with 2n=48-56 (Reig *et al.*, 1992). This group is formed by seven species: *C. mendocinus* (2n=47-48), *C. azarae* (2n=46-48), *C. chasiquensis* (2n=47-48), *C. rionegrensis* (2n=48-56), *C. porteousi* (2n=47-48), *C. australis* (2n=48), and *C. flamarioni* (2n=48) (Massarini *et al.*, 1991; Reig *et al.*, 1992; Freitas, 1994; Massarini *et al.*, 1998; D’Elia *et al.*, 1999; Massarini and Freitas, 2005). All species from this group present the asymmetric sperm form (Vitullo *et al.*, 1988; Freitas, 1994, 1995; Massarini *et al.*, 1998). The *mendocinus* group is found in centralwestern Argentina, western Uruguay, and in the coastal plain of southern Brazil (Massarini *et al.*, 1991, Massarini and Freitas, 2005) (Figure 1).

The *torquatus* group, proposed by Parada *et al.* (2011), shows a high chromosomal diploid number variation, from 2n=40 to 2n=70. It is formed by *C. torquatus* with 2n=40-46 (Freitas and Lessa, 1984; Fernandes *et al.*, 2009a,b), *C. lami* with 2n=54-58 (Freitas, 2001, 2007), *C. minutus* with 2n=42-50 (Freitas, 2006), *C. perrensi* with 2n=50-58 (Ortells *et al.*, 1990, Reig *et al.*, 1992), *C. pearsoni* with 2n=56-70 (Novello and Altuna, 2002), *C. roigi* with 2n=48 (Ortells *et al.*, 1990), and *C. ibicuiensis* with 2n=50 (Freitas *et al.*, 2012). All species from this group present symmetric sperm form (Vitullo *et al.*, 1988; Freitas, 1995). The *torquatus* group occurs in Northern and Southern Uruguay, Southern Brazil, and Northeastern Argentina (Freitas, 1994, 2006; Parada *et al.*, 2011) (Figure 1).

Both groups occupy heterogeneous habitats, from dunes in the Atlantic coast to low valleys in the West (Reig *et al.*, 1990) (Figure 1). Molecular phylogenetic analyses support the *mendocinus* and *torquatus* groups as two monophyletic clades (D’Elia *et al.*, 1999; Castillo *et al.*, 2005; Parada *et al.*, 2011). Contreras and Bidau (1999) proposed that chromosomal rearrangements could play an important role in the source of reproductive isolation in small populations (common in several species of genus *Ctenomys*). Thus, if chromosomal rearrangements act in reproductive isolation and allow populations to evolve independently of each other (by natural selection or genetic drift), we expected that the *torquatus* group, which has high chromosomal polymorphism, would show more variable skull shapes and sizes than the *mendocinus* group, which has low chromosomal polymorphism.

Geometric morphometrics is more efficient in capturing information related to the shape of the organisms and presents a greater statistical robustness than traditional measurements. In addition, it allows for the reconstruction of changes in shape and statistical inference, which is very important for the visualization of shape differences (Rohlf and Marcus, 1993). Some studies used the geometric morphometric approach to investigate the relationship between chromosomal polymorphism and morphological skull variation in *Ctenomys* at an intraspecific level (Fernandes *et al.*, 2009a; Fornel *et al.*, 2010). Therefore, at the interspecific level (among species) there is a lack of information on the role of chromosomal rearrangements in morphological evolution of *Ctenomys*.

Much controversy remains on the role of chromosomal diploid number variation related to speciation in the genus *Ctenomys*. Therefore, the aim of this study was to investigate the variation in skull shape and size within and between the *mendocinus* and *torquatus* groups and test the association of chromosomal polymorphism and skull morphological variation in these two groups.

## Material and Methods

### Sample

We analyzed 763 skulls and 515 mandibles of adults representing 12 species from the *mendocinus* and *torquatus* groups (Table 1). The skulls and mandibles were obtained from the following museums and scientific collections: Departamento de Genética, Universidade Federal do Rio Grande do Sul, Porto Alegre, Brazil (UFRGS); Museo Nacional de História Natural y Antropología, Montevideo, Uruguay (MUNHINA); Museo Argentino de Ciencias Naturales “Bernardino Rivadavia”, Buenos Aires, Argentina (MACN); Museo de La Plata, La Plata, Argentina (MLP); Museo de Ciencias Naturales “Lorenzo Scaglia”, Mar del Plata, Argentina (MMP); Museum of Vertebrate Zoology, University of California, Berkeley, USA (MVZ); American Museum of Natural History, New York, USA (AMNH); and Field Museum of Natural History, Chicago, USA (FMNH). We assumed that sexual dimorphism was negligible for the present study. Interspecific differences are in general greater than sexual differences, so we used males and females together in all analyses.

**Table 1:** Sample size of skulls and mandibles of 12 species of *Ctenomys* from *mendocinus* and *torquatus* groups.

Species	Group	N_Skull_	N_Mandible_
*C. australis* (aus)	*mendocinus*	31	27
*C. azarae* (aza)	*mendocinus*	29	26
*C. flamarioni* (fla)	*mendocinus*	32	22
*C. porteousi* (por)	*mendocinus*	30	28
*C. mendocinus* (men)	*mendocinus*	24	14
*C. ibicuiensis* (ibi)	*torquatus*	16	10
*C. lami* (lam)	*torquatus*	89	66
*C. minutus* (min)	*torquatus*	197	122
*C. pearsoni* (pea)	*torquatus*	77	60
*C. perrensi* (per)	*torquatus*	9	9
*C. roigi* (roi)	*torquatus*	7	7
*C. torquatus* (tor)	*torquatus*	222	124
Total		763	515

### Geometric morphometrics

**Figure 2:**
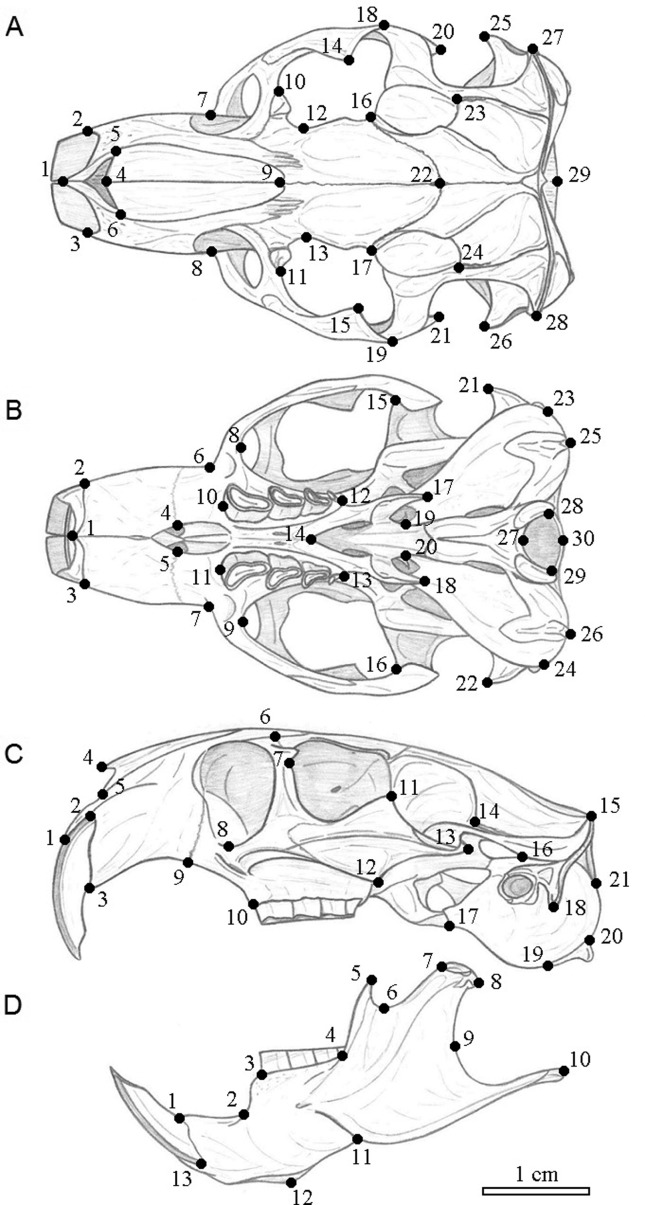
Landmarks location on skull of *Ctenomys* for dorsal (A), ventral (B), and lateral (C) views of the cranium and lateral view of the mandible (D). See Table S1 for anatomical description of each landmark.

Each cranium was photographed in the dorsal, ventral, and left lateral views of the skull and on the left side of the mandible with a digital camera, at a resolution of 3.1 megapixels (2048 × 1536), using the macro function without flash. We used 29 two-dimensional landmarks for dorsal, 30 for ventral, and 21 for lateral views of the skull, as proposed by Fernandes *et al.* (2009a), though Fornel *et al.* (2010) added another 13 landmarks for the lateral view of the mandible (Figure 2; Supplementary Table S1). Anatomical landmarks were positioned for each specimen using TPSDig version 1.40 software (Rohlf, 2004). All landmarks were captured by the same person (R.F.). Coordinates were superimposed using a generalized Procrustes analysis (GPA) algorithm (Dryden and Mardia, 1998), since GPA removes differences unrelated to the shape, such as scale, position, and orientation (Rohlf and Slice, 1990; Rohlf and Marcus, 1993; Bookstein, 1996a, 1996b; Adams *et al.*, 2004). We symmetrized landmarks on both sides of the skull’s dorsal and ventral views, and only the symmetric part of the variation was analyzed (Kent and Mardia, 2001; Klingenberg *et al.*, 2002; Evin *et al.*, 2008). The size of each skull was estimated using its centroid size, the square root of the sum of squares of the distance of each landmark from the centroid (mean of all coordinates) of the configuration (Bookstein, 1991).

### Statistical analysis

For testing skull size differences we used analysis of variance (ANOVA) of the centroid size. For multiple comparisons of centroid size, we used Tukey’s test and Box plots to visualize its variation. For skull shape we used principal component analysis (PCA), canonical variate analysis (CVA), and multivariate analysis of variance (MANOVA) of the principal components (PCs). To choose the number of PCs to be included in the linear discriminant analysis (LDA), we computed correct classification percentages with each combination of PCs (Baylac and Friess, 2005). We selected the subset of PCs giving the highest overall correct classification percentage. We then used a leave-one-out cross validation procedure that allows for an unbiased estimate of classification percentages (Ripley, 1996; Baylac and Friess, 2005). Cross validation is used to evaluate the performance of classification by LDA. We used LDA for computed correct classification percentages among groups and species. The Mahalanobis’s *D* ^2^ distances were used to generate phenograms with the neighbor-joining method. Finally, we used Procrustes distances to measure the variation in skull shape within the *mendocinus* and *torquatus* groups, and used Levene’s test to assess the equality of variances in different groups.

For all statistical analyses, as well as for generating graphs we used the R language and environment version 2.9.0 for Windows (R Development Core Team, http://www.R-project.org) and the following libraries: MASS (Venables and Ripley, 2002), ape version 1.8-2 (Paradis *et al.*, 2004), stats (R Development Core Team), and ade4 (Dray and Dufour, 2007). Geometric morphometric procedures were carried out using the Rmorph package, a geometric and multivariate morphometrics library for R (Baylac, 2008).

## Results

### Size

**Figure 3:**
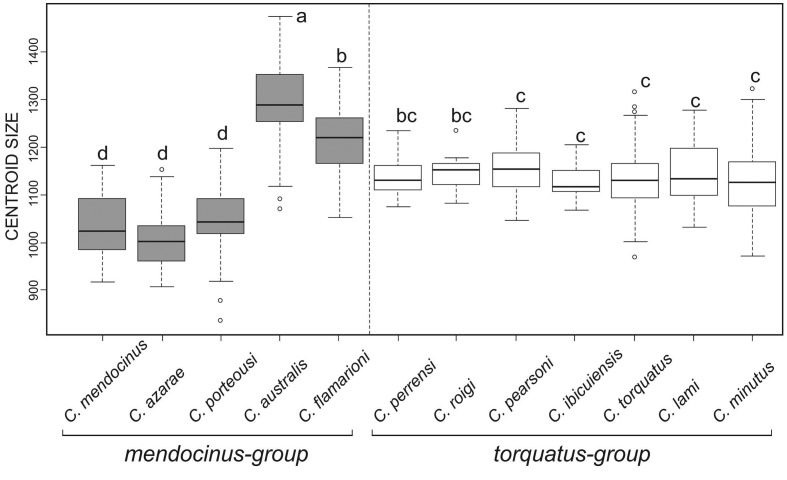
Skull centroid size variability among 12 species of *Ctenomys* from the *mendocinus* and *torquatus* groups for dorsal view of the skull. The horizontal line represents the median, box margins are at the 25th and 75th percentiles, bars extend to 5th and 95th percentiles, and circles are outliers. Different letters above boxes represent significant differences among species for Tukey’s multiple comparison tests at the 5% level.

The two groups, *mendocinus* and *torquatus*, did not differ significantly in skull centroid size (*P* > 0.05). We found significant differences among species for size (dorsal: *F* = 42.94, *P* < 0.001; ventral: *F* = 39.24, *P* < 0.001; lateral: *F* = 38.96, *P* < 0.001; and mandible: *F* = 38.7, *P* < 0.001). However, the Tukey test showed no significant difference among species belonging to the *torquatus* group (*P*> 0.05) (Figure 3). The species from the *mendocinus* group were more varied in skull centroid size than the *torquatus* group, with *C. australis* being significantly bigger than the other species in both groups (Tukey: *P* < 0.001 in all pairwise comparisons) (Figure 3).

### Shape – two groups

**Figure 4:**
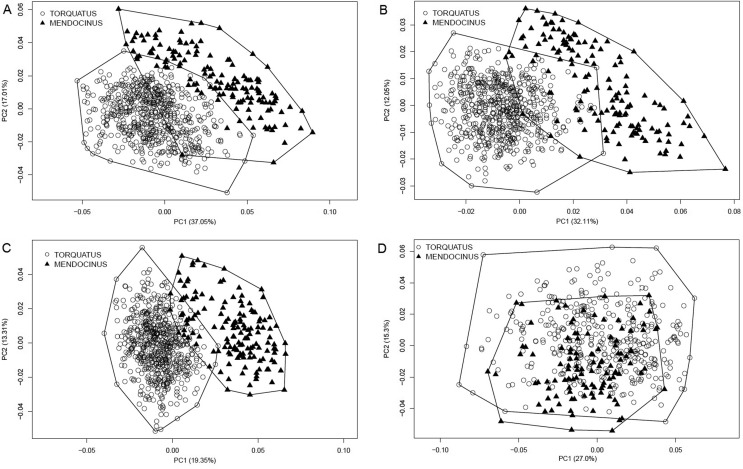
Scatterplot of principal component analysis (PCA) show the two first PCs for two groups of *Ctenomys*, the *mendocinus* and *torquatus* groups for dorsal (A), ventral (B), and lateral (C) views of the skull and lateral view of the mandible (D). Variance percentages for PC1 and PC2 are given in parenthesis.

PCA for the three views of the cranium showed two structured groups with low superimposition corresponding to the *mendocinus* and *torquatus* groups (Figure 4A-C). Regarding the mandible, there was no difference between groups (Figure 4D). The LDA for three views of the skull and mandible showed higher percentages of correct classification for the *torquatus* group (Table 2). The lateral view of the skull had the highest (100%) and the mandible the lowest (94.87% for *mendocinus* and 97.16% for *torquatus*) percentage of correct classification in LDA (Table 2). Comparison between the two groups was significant for all views of the skull (dorsal: λ_Wilks_ = 0.17, *F* = 365.4, *P* < 0.001; ventral: λ_Wilks_ = 0.18, *F* = 246.68, *P* < 0.001; lateral: λ_Wilks_ = 0.21, *F* = 555.57, *P* < 0.001; and mandible: λ_Wilks_ = 0.31, *F* = 84.22, *P* < 0.001).

**Table 2:** Percentage of correct classification for *mendocinus* and *torquatus* groups using linear discriminant analysis (LDA) for dorsal, ventral, and lateral views of the skull, and lateral view of the mandible.

	Group	
	*mendocinus*	*torquatus*
Dorsal	99.31	100
Ventral	98.63	100
Lateral	100	100
Mandible	94.87	97.16

**Figure 5:**
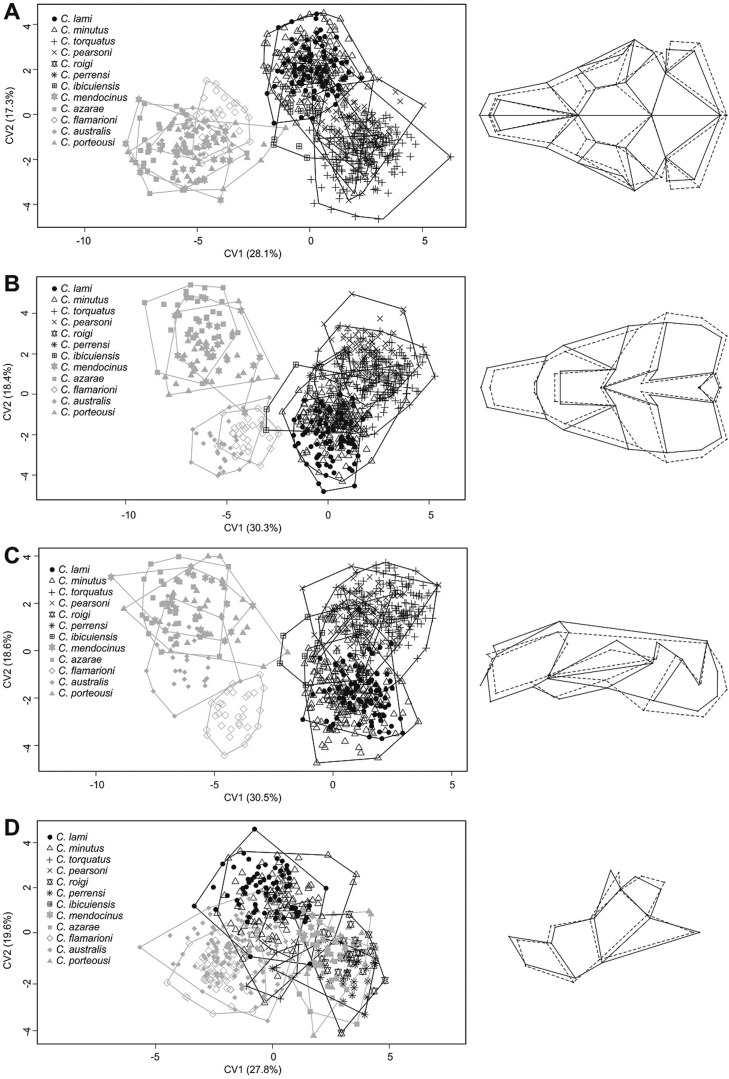
Scatterplot of canonical variate analysis (CVA) show the two first canonical axis for 12 species of *Ctenomys* from the *mendocinus* and *torquatus* groups for dorsal (A), ventral (B), and lateral (C) views of the skull and lateral view of the mandible (D). The grids at the right side of each plot represent the differences for landmark configuration along the first CV, where dotted lines represent the extreme negative scores and solid lines represent the extreme positive scores. Variance percentages for CV1 and CV2 are given in parenthesis.

Skull shape differences between the two groups and among species are given in a CVA scatterplot (Figure 5). In the *mendocinus* group, the skull’s three views provided similar results, while the *torquatus* group showed separation in the 1^st^ canonical axes (Figure 5A-C). The *torquatus* had a proportionally bigger rostrum, larger zygomatic arch, deeper skull, and a proportionally larger coronoid process in the mandible than the *mendocinus* (Figure 5A-C). The *mendocinus* group animals have longer nasals and a larger tympanic bulla than those of the *torquatus* group (Figure 5A-C). For the mandible, CVA did not show separation between the two groups (Figure 5D).

### Shape – species

There was a significant difference among species (dorsal: λ_Wilks_ = 0.002, *F* = 30.41, *P* < 0.001; ventral: λ_Wilks_= 0.002, *F* = 32.8, *P* < 0.001; lateral: λ_Wilks_ = 0.002, *F*= 33.33, *P* < 0.001; mandible: λ_Wilks_ = 0.017, *F* = 16.68, *P*< 0.001).

The LDA for dorsal views of the skull showed the highest percentage of correct classification for *C. australis*, *C. flamarioni*, and *C. roigi* (100%, Table 3). The species *C. mendocinus* and *C. perrensi* showed the lowest values of correct classification (75% and 77.7%, respectively, Table 3). Almost all specimens were classified in the correct group, the only exception being *C. porteousi*, which belongs to the *mendocinus* group and had two individuals classified erroneously in the *torquatus* group (Table 3). The other views of the skull and mandible showed lower percentages of classification than the dorsal view of the skull (data not shown).

**Table 3:** Classification of 12 species of *Ctenomys* from *mendocinus* and *torquatus* groups for dorsal view of the skull using linear discriminant analysis (LDA). The diagonal line shows the samples that were correctly classified. The percentage of correct classification is given in the last line. The species abbreviations follow the same order in the first column and Table 1.

Group	*mendocinus*					*torquatus*						
Species	*aus*	*aza*	*fla*	*por*	*men*	*lam*	*min*	*pea*	*per*	*roi*	*tor*	*ibi*
*C. australis*	31											
*C. azarae*		28		1								
*C. flamrioni*			32									
*C. porteousi*	1	2		24	1		2					
*C. mendocinus*		2			18							
*C. lami*						78	11					
*C. minutus*						6	186	2			3	
*C. pearsoni*							1	66			10	
*C. perrensi*									7	1	1	
*C. roigi*										7		
*C. torquatus*							3	4	1		214	
*C. ibicuiensis*							1				1	14
Percentage	100	96.6	100	80	75	87.6	94.4	85.7	77.7	100	96.4	87.5

**Figure 6:**
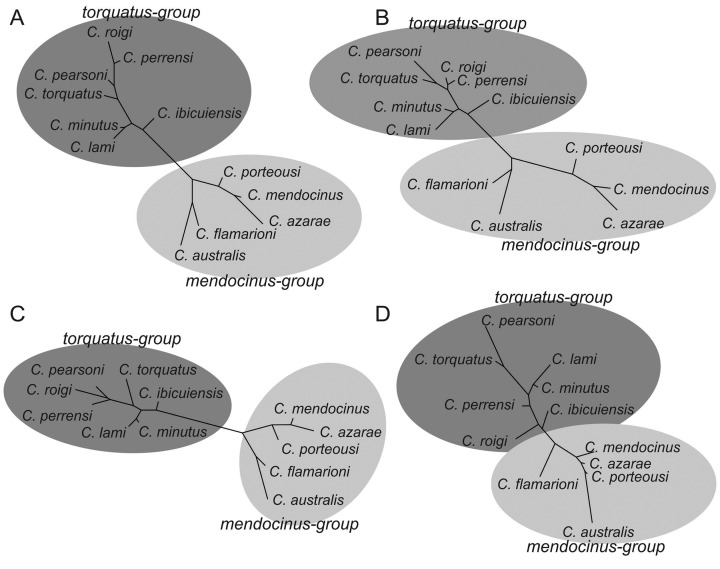
Phenogram using neighbor-joining method and Mahalanobis distances from lateral view of the skull for 12 species of *Ctenomys* from the *mendocinus* and *torquatus* groups.

The phenogram using morphological data for dorsal, ventral, and lateral views of the skull showed a larger separation between *mendocinus* and *torquatus* groups (Figure 6A,B,D) than those of the mandible (Figure 6D). Moreover, the Mahalanobis distances in the cladogram indicate a subdivision in the *mendocinus* group, with a strong morphological association between *C. australis* and *C. flamarioni*, separated from *C. mendocinus*, *C. porteousi*, and *C. azarae* (Figure 6). In the same way, in the *torquatus* group, *C. lami* and *C. minutus* were strongly associated.

### Intragroup variation

The variation amplitude of Procrustes distances did not differ significantly between the *mendocinus* and *torquatus* groups for dorsal, ventral, and lateral views of the skull and mandible (Levene’s test: *F* = 0.221, *P* = 0.64; *F* = 0.005, *P* = 0.94; *F* = 0.083, *P* = 0.77; *F* = 0.082, *P* = 0.78, respectively).

## Discussion

We analyzed skull and mandible shape and size variation within and between the *mendocinus* and *torquatus* groups of the genus *Ctenomys*. Our results agree with other studies in determining that the two groups have very different skull morphologies. There is no evidence of convergence among species from different groups.

Contreras and Bidau (1999) suggested that chromosomal rearrangements could reduce gene flow and even promote isolation among populations. Nevertheless, some works demonstrated the occurrence of hybrid zones between different chromosomal rearrangements (Freitas, 1997; Gava and Freitas, 2002, 2003). Moreover, Fernandes *et al.* (2009a) found that chromosomal evolution and phenotypic variation are not necessarily related. We reject the hypothesis that a high chromosomal polymorphism is associated to a high morphological variation at the interspecific level in the *mendocinu* group. Our data showed that besides *mendocinus* and *torquatus* groups displaying very different chromosomal polymorphisms, there is no evidence of association between chromosomal diploid number and skull shape variation. Rieseberg and Livingstone (2003) proposed that the reduced recombination of rearranged chromosomes might favor the accumulation of adaptive differences on rearranged regions. Our data did not support this hypothesis in the genus *Ctenomys*, because the *mendocinus* group with low chromosomal polymorphism showed skull shape variation (amplitude of variation) like the *torquatus* group, which presented high chromosomal polymorphism. These results agree with Tomasco and Lessa (2007) who argue that chromosomal speciation might not be the main factor in *Ctenomys* diversification.

The *mendocinus* group occurs in heterogeneous habitats, from coastal dunes to the proximity of the Andes. Several species of *Ctenomys* are characterized as scratch (claws) and chisel-tooth (incisors) diggers. These incisors can be used for building tunnel systems, and soil hardness could influence the incisor procumbency and affect skull morphology (Vassallo, 1998; Mora *et al.*, 2003; Verzi and Olivares, 2006). In the species of the *mendocinus* group we found a pattern in skull centroid size. Populations near the ocean coasts are bigger than those inland (see Figures 1 and 3). Thus, different types of soil hardness could play a role in biomechanical constraints and diversification in skull morphology of the *Ctenomys*: smaller skulls for hard soils and lager skulls for soft soils. Nevertheless, this size difference between regions could affect skull morphology due to different dietary types. *Ctenomys* are herbivorous and feed on a variety of grasses, eating both the subterranean and subaereal parts of gramineae (Reig *et al.*, 1990; Lopes *et al.*, 2015). Thus, primary productivity, food quality, and abundance may influence body size (Medina *et al.*, 2007). Nevertheless, we do not have knowledge on the ecology of all *mendocinus* group species, such as data about vegetable richness, in order to completely explain the difference in skull size. The *mendocinus* group occupies a larger area than the *torquatus* group and its distribution is more fragmented (Figure 1). This more intensive isolation of the *mendocinus* species could allow for a larger differentiation among them. In this regard, we found a strong association between *C. australis* and *C. flamarioni*: both are found in the sand dunes of the Atlantic coast and are more distant from other *mendocinus* species (Figure 6). Thus, both ecologic and phylogenetic constraints permit *C. australis* and *C. flamarioni* to be very close.

Mandible shape and size were less variable than skull in the two *Ctenomys* groups, making for a rather weak discriminatory structure. A more confined amount of morphological variation was observed in the mandible of *C. minutus* (Fornel *et al.*, 2010). This is probably the result of stabilizing selection, since the functions of the mandible are more restricted than in the rest of the skull (Borges *et al.*, 2017).

Medina *et al.* (2007) found that the genus *Ctenomys* follows the converse of Bergmann’s rule. This agrees with our data: larger species occupy warm areas while smaller species occupy cold and inner continent areas near the Andes mountain range. Thus, thermoregulation may not be a great constraint to subterranean species, because tunnel systems protect from the outside weather.

New studies on the association between morphological and geographical distances and on several aspects of ecological, demographic, as well as historical factors of the different *Ctenomys* species will help understand the evolution and the explosive cladogenesis seen in this group of rodents in Neotropical regions.

## Supplementary material

The following online material is available for this article:

Table S1 - Definition of landmarks.
