# Supplementary material for: Skull shape and size variation within and between mendocinus and torquatus groups in the genus Ctenomys (Rodentia: Ctenomyidae) in chromosomal polymorphism context
Source: Genet Mol Biol. 2018;41(1 Suppl 1):263–72. doi: 10.1590/1678-4685-GMB-2017-0074 (PMC5913726; doi:10.1590/1678-4685-GMB-2017-0074)
Supplement: Supplementary file 1 [file 1415-4757-GMB-41-01-2017-0074-s001.pdf]

Supplementary Material to "Skull shape and size variation within and between mendocinus and torquatus groups in the genus Ctenomys (Rodentia: Ctenomyidae) in chromosomal polymorphism context"

Table S1 - Definition of landmarks**.** Numbers and locations of landmarks in each view of the cranium and lower jaw of *Ctenomys* (represented in Figure 2)

Position	Landmarks
Dorsal view of the cranium	1. anterior tip of the suture between premaxillas; 2-3. anterolateral extremity of incisor alveolus; 4. anterior extremity of the suture between nasals; 5-6. anteriormost point of suture between nasal and premaxilla; 7-8. Anterior most point of the root of zygomatic arch; 9. suture between nasals and frontals; 10-11. anterolateral extremity of lacrimal bone; 12-13. point of least width between frontals; 14-15. tip of extremity of superior jugal process; 16-17. anterolateral extremity of suture between frontal and squamosal; 18-19. lateral extremity of suture between jugal and squamosal; 20-21. tip of posterior process of jugal; 22. suture between frontals and parietals; 23-24. anterolateral extremity of suture between parietal and squamosal; 25-26. anterior tip of external auditory meatus; 27-28. point of maximum curvature on mastoid apophysis; 29. posteriormost point of occipital along the midsagittal plane
Ventral view of the cranium	1. anterior tip of suture between premaxillas; 2-3. anterolateral extremity of incisor alveolus; 4-5. lateral edge of incisive foramen in suture between premaxilla and maxilla; 6-7. anteriormost point of root of zygomatic arch; 8-9. anteriormost point of orbit in inferior zygomatic root;10-11. anteriormost point of premolar alveolus; 12-13. posterior extremity of III molar alveolus; 14. posterior extremity of suture between palatines; 15-16. anteriormost point of intersection between jugal and squamosal; 17-18. posteriormost point of pterygoid; 19-20. anterior extremity of tympanic bulla; 21-22. anterior tip of external auditory meatus; 23-24. posterior extremity of mastoid apophysis; 25-26. posterior extremity of paraoccipital apophysis; 27. anteriormost point of foramen magnum; 28-29. posterior extremity of occipital condyle in foramen magnum; 30. posteriormost point of foramen magnum along midsagittal plane
Lateral view of the cranium	1. anteriormost point of premaxilla; 2. posteriormost point of incisor alveolus; 3. inferiormost point of incisor alveolus; 4. anterior tip of nasal; 5. anteriormost point of the suture between nasal and premaxilla; 6. suture between premaxilla, maxilla and frontal in superior zygomatic root; 7. inferiormost point of suture between lacrimal and maxilla; 8. inferiormost point of infraorbital foramen in inferior zygomatic root; 9. inferiormost point of suture between premaxilla and maxilla; 10. anteriormost point of premolar alveolus; 11. extremity of superior jugal process; 12. extremity of inferior jugal process; 13. tip of posterior jugal process; 14. medial point of suture between parietal and squamosal; 15. superior extremity of lambdoidal crest; 16. posterior extremity of postglenoid fossa; 17. inferior extremety in suture between pterygoid and tympanic bulla; 18. inferior extremity of mastoid apophysis; 19. anteriormost margin of paraoccipital apophysis; 20. posteriormost margin of paraoccipital apophysis; 21. posterior extremity of intersection between occipital and tympanic bulla
Lateral view of the mandible	1. upper extreme anterior border of incisor alveolus; 2. extreme of the diastema invagination; 3. anterior edge of the premolar alveolus; 4. intersection between molar alveolus and coronoid process; 5. tip of the coronoid process; 6. maximum of curvature between the coronoid and condylar processes; 7. anterior edge of the articular surface of the condylar process; 8. tip of the postcondyloid process; 9. maximum curvature between condylar and angular processes; 10. tip of the angular process; 11. intersection between mandibular body and masseteric crest; 12. posterior extremity of the mandibular symphysis; 13. posterior extremity border of incisor alveolus
